# Editorial: Zeolite Chemistry and Applications

**DOI:** 10.3389/fchem.2020.00118

**Published:** 2020-03-06

**Authors:** Marcelo M. Pereira, Benoit Louis, Qiang Wang

**Affiliations:** ^1^Departamento de Química Inorgânica, Instituto de Química, Universidade Federal do Rio de Janeiro, Rio de Janeiro, Brazil; ^2^ICPEES - Institut de Chimie et Procédés pour l'Energie, l'Environnement et la Santé, Energy and Fuels for a Sustainable Environment Team, UMR 7515 CNRS - Université de Strasbourg - ECPM, Strasbourg, France; ^3^Environmental Functional Nanomaterials (EFN) Laboratory, College of Environmental Science and Engineering, Beijing Forestry University, Beijing, China

**Keywords:** zeolites, porous-material, bi-functional catalysts, environmental, green fuel

A better future for humanity demands improved chemical processes that are more efficient and able to use several types of feedstocks, with less environmental damage. In addition, the chemical industry must provide essential goods and desired products. Catalysts can bridge the gap to solving these issues. Thus, improvement in catalyst properties, new routes for catalyst preparation, and new catalysts will be key to impacting the development of green energy, efficient fuel production, and environmental remediation.

Zeolites, porous material, and bi-functional catalysts represent a significant market. They alone in the world respond almost entirely to fuel production, and besides being also relevant to petrochemical and pharmaceutical industries, contribute to decreasing greenhouse gas emission and environmental remediation. The present edition of “Zeolite Chemistry and Applications” explores not only regular but also new catalysts in current chemical and new processes. This edition contains 12 articles, mainly in three areas: (1) green fuel production, (2) environmental remediation, and (3) catalyst technology.

The first area includes the production of green hydrocarbons by introducing biomass in a typical refinery. In this article, it was shown that faujasite zeolite converts a ketal-sugar derivative efficiently into green-aromatics (Pinto et al.). A mini review article explores the direct methane conversion into methanol in the presence of a copper zeolite catalyst. The authors show that the type of zeolite is crucial to obtaining a high methanol yield, i.e., small pores have enabled higher methanol yields than medium and large pore Cu-zeolites (Park et al.). Methane can be produced from both natural gas and bio-methane, and methanol is a platform molecule for sequential conversion. Thus, the route methane-methanol fits the requirement of green or friendly fuel production. Furthermore, methanol conversion into benzene, toluene, and xylene (BTX) was carried out in the presence of a b-oriented ZSM-5 zeolite film. This film was synthesized on the macropore α-quartz substrate, modified with titanium dioxide (TiO_2_), polyvinyl acetate (PVA), and chitosan (CTS) (Chu et al.). The modification improves the b-oriented ZSM-5 zeolite film; as TiO_2_ coating provides a smooth nucleus-substrate growth interface for the ZSM-5 zeolite film, it leads to a uniform distribution of the ZSM-5 zeolite film growing along the b-oriented direction. Another way to modify catalyst properties is the preparation of zeolites on the carbons template (Molefe et al.). The metal-organic frameworks (MOFs) and zeolite template carbons (ZTC) showed a remarkably higher area (1,054–2,433 m^2^ g^−1^) with higher hydrogen uptake capacity (1.22–1.87 H_2_ wt.%, respectively at 1 bar and 77 K). Finally, a series of ZSM-5 zeolites with hierarchical porous structures were synthesized using NaOH solutions treatment method. Herein, the hierarchical ZSM-5 was obtained with a uniform size of the mesoporous and microporous structure and high acid sites, and as a consequence produced more adsorption sites and thus increased the adsorption of benzene (Meng et al.).

The second area explores environmental remediation by trapping organic compounds produced by regular processes, the carbon industry, and carbon dioxide. One article explores the hierarchical concept in MOFs of benzene adsorption. Novel hierarchical Fe(III)-doped Cu-MOFs (Fe-HK) were developed via the introduction of Fe^3+^ ions during HKUST-1 synthesis. It was found that the hierarchical-pore Fe-HK-2 exhibited optimal textural properties with a highly BET surface area (1,707 m^2^/g) and a total pore volume of 0.93 cm^3^/g, which were higher than those of the unmodified HKUST-1. Furthermore, the novel material showed enhanced diffusion rate and excellent reversibility of benzene adsorption. The outstanding adsorption capacity of benzene showed by this novel material might be an attractive candidate for volatile organic compounds (VOCs) adsorption (Sun et al.). In another paper, the trap of VOCs (by adsorption-desorption) was carried out in the presence of hierarchical composites of ZSM-5 with MCM-41 and Silicalite (Li et al.). These catalysts were prepared for improving hydrophobicity by applying different optimized coating dosages (25, 50, and 75%) on designed novel ZSM-5/MCM-41 and ZSM-5/Silicalite-1 hierarchical composites. The relatively large specific surface area and pore volume of the ZSM-5/MCM-41 composite presented a longer toluene adsorption time no matter if in dry or 50% humid conditions. In another paper, Zeolite X was prepared by using aluminum Residue from Coal Fly Ash (Zhu et al.). The adsorption of isopropanol, benzene, and cyclohexane was carried out in zeolite as prepared, and values around 15% (in wt.% of VOCs/wt.% zeolite) were obtained. It is important to point out the advantage of this method in terms of both environmental friendliness and economic viability. Further, for a different application, zeolites, layered double hydroxides (LDH), and zeolites-coated LDH composites were prepared and used for CO_2_ trapping (by adsorption) (Megías-Sayago et al.). Those materials demonstrated good CO_2_ sorption capacity (>1 mmol of CO_2_/g of material at 40°C). CO_2_ capture strongly depends on the properties of the material.

One example of a bi-functional catalyst was found by adding vanadium (V) oxides onto the surface of NaZSM-5 zeolites (V/ZSM), then applying this to the removal of recalcitrant organic chemicals (ROCs) from the water by ozonation treatment (Xu et al.). The novel catalytic materials were found to be highly efficient in the removal of the nitrobenzene and benzoic acid archive, achieving 100% degradation in ~15 min. The key for such a goal was the highly dispersed V oxides (V4+ and V5+) and Si-OH-Al framework structures that promote the surface reaction and generation of hydroxyl radicals. Another example of a new bi-functional material was also presented in this issue. Besides catalysis, loading of active metals and metal nanoparticles in Metal-Organic Frameworks (MOFs) is an emergent field, with applications in sensors, medicine, and even in the polymeric industry. It was shown that Ag nanoparticles of a size of around 2.5 nm appear inside the MOF pores periodically, as well as in the particle distribution with a much larger size (of ca. ≈13 nm of diameter) observed outside the framework. (Mahugo et al.). This work makes clear that simple experimental modifications can have a significant influence on both chemistry and the location of the Ag species. More importantly, the advanced TEM techniques developed in this study have shown enormous potential to characterize metal particles on several porous materials. Thus, it is expected that this approach will be used in several applications. Another important theme is the modification of a catalyst surface, enabling better stabilization of blended catalysts. A beautiful example of a new route for catalyst preparation is described in the article (Wu et al.), which worked on the structure-absorption relationship of common surface modifiers of chitosan (CTS), polyvinyl acetate (PVA), and titanium dioxide (TiO_2_) with α-quartz surface using molecular dynamics simulation. For example, the formation of several stable Ti-O-Si chemical bonds (between the TiO_2_ and the surface of α-quartz) stabilizes the attached ZSM-5 film. Thus, several applications could emerge for this new type of catalyst.

To summarize, the present edition of “Zeolite Chemistry and Applications” showed examples that link the properties of zeolites and porous materials with application problems. It is impressive that one can find in these 12 articles very relevant questions on catalysis with broad applications. Let's wait to see the catalysts and related materials—the heart of chemical processes—contribute to a better future for humanity. This issue is a small sample of this potential.

## Author Contributions

MP, BL, and QW contributed equally to this article and have made a substantial, direct and intellectual contribution to the work, and approved it for publication.

### Conflict of Interest

The authors declare that the research was conducted in the absence of any commercial or financial relationships that could be construed as a potential conflict of interest.

